# Determination of exciton binding energy using photocurrent spectroscopy of Ge quantum-dot single-hole transistors under CW pumping

**DOI:** 10.1038/s41598-023-41582-8

**Published:** 2023-08-31

**Authors:** Po-Yu Hong, Chi-Cheng Lai, Ting Tsai, Horng-Chih Lin, Thomas George, David M. T. Kuo, Pei-Wen Li

**Affiliations:** 1 Institute of Electronics, National Yang Ming Chiao Tung University, Hsinchu, Taiwan; 2 Department of Electrical Engineering, National Central University, Chungli, Taiwan

**Keywords:** Electrical and electronic engineering, Single photons and quantum effects, Single photons and quantum effects

## Abstract

We reported exciton binding-energy determination using tunneling-current spectroscopy of Germanium (Ge) quantum dot (QD) single-hole transistors (SHTs) operating in the few-hole regime, under 405–1550 nm wavelength (λ) illumination. When the photon energy is smaller than the bandgap energy (1.46 eV) of a 20 nm Ge QD (for instance, λ = 1310 nm and 1550 nm illuminations), there is no change in the peak voltages of tunneling current spectroscopy even when the irradiation power density reaches as high as 10 µW/µm^2^. In contrast, a considerable shift in the first hole-tunneling current peak towards positive V_G_ is induced (ΔV_G_ ≈ 0.08 V at 0.33 nW/µm^2^ and 0.15 V at 1.4 nW/µm^2^) and even additional photocurrent peaks are created at higher positive V_G_ values (ΔV_G_ ≈ 0.2 V at 10 nW/µm^2^ irradiation) by illumination at λ = 850 nm (where the photon energy matches the bandgap energy of the 20 nm Ge QD). These experimental observations were further strengthened when Ge-QD SHTs were illuminated by λ = 405 nm lasers at much lower optical-power conditions. The newly-photogenerated current peaks are attributed to the contribution of exciton, biexciton, and positive trion complexes. Furthermore, the exciton binding energy can be determined by analyzing the tunneling current spectra.

## Introduction

Single-electron or single-hole transistors (SETs/SHTs), comprising a single QD capacitively coupled to source/drain reservoirs and plunger-gates through tunneling barriers and gate-dielectric layers, respectively, are the ultimate embodiment for electronic devices controlling tunneling current with *single-charge* precision based on Coulomb blockade effects. Their inherent charge-number distinguishability makes QD-SETs (or SHTs) an unrivaled readout device for charge- and spin-qubits in terms of charge-sensing and spin-to-charge conversion, respectively^1–7^. Thanks to their high charge sensitivity, both SETs and SHTs are also anticipated to be highly sensitive for photodetection. Once photons are absorbed, photogenerated electron–hole pairs result in changes in the differential conductance and tunneling-current spectroscopy of SETs/SHTs^8–12^. Besides, the large peak-to-valley current ratio (PVCR) of SHTs at room temperature suggests that SHTs are able to suppress noise from other high-level excitations^13,14^. Therefore, SHT-based photodetectors offer advantages of high sensitivity and low noise. Additionally, the hole-hole charging energy (*U*_*hh*_) is larger than the electron–electron charging energy (*U*_*ee*_) since holes have a larger effective mass than electrons. Consequently, it would be easier for SHTs to distinguish tunneling-current spectra involving biexciton and exciton transport processes^12^.

Thanks to the advancements in CMOS fabrication technology, the operation of SHTs in the few-charge regime has been experimentally demonstrated using small Si QDs^13^ or Ge QDs^14–18^. Ge-QD SHTs are particularly attractive because Ge QDs are more likely to have a pseudo-direct bandgap structure for better photon-charge conversion than Si QDs, due to a larger exciton Bohr radius (α_B_) of 24 nm in Ge than in Si (α_B, Si_ = 4.9 nm). Our previous work has already reported experimental fabrication and steady-state transfer characteristics (I_D_-V_G_) of Ge-QD SHTs, comprising a single Ge spherical QD (20 nm in diameter) self-aligned with source/drain reservoirs of boron-doped Si via tunneling barriers of SiO_2_/Si_3_N_4_^17^. Experimental observation of aperiodic oscillatory peaks with large PVCR (> 100) and current plateaus with negative differential conductance at *T* = 4 – 40 K evidences our Ge-QD SHTs operating in the few-hole regime. Large single-hole addition energies of > 100 meV and ~ 50 meV for hole number changing from *N* = 0 → 1 and 1 → 2, respectively, were extracted from the slopes of Coulomb diamonds^17^. In this work, we advanced the exploration of our Ge QD-SHTs for exciton binding-energy determination by studying photoexcitation effects on tunneling current spectroscopy under continuous-wave laser irradiations at wavelengths (λ) of 400–1550 nm. We observed that photons with energies greater than 1.45 eV are able to excite additional photocurrent peaks at more positive gate voltages (V_G_ = − 0.775 V and − 0.6 V/− 1.01 V) with respect to the first/second tunneling current peaks (at V_G_ = − 0.82 V/− 1.23 V) corresponding to the single-hole/two-hole states measured in the darkness. Irradiated power effect on the intensity and position of newly generated photocurrent peaks were studied.

## Results

**Figure 1:**
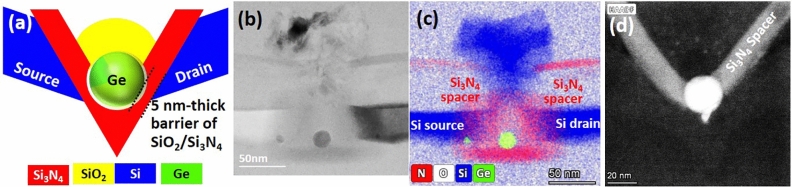
(**a**) Schematic diagram, cross-sectional, (**b**) TEM/(**c**) EDS mapping and (**d**) plan-view STEM micrographs of studied Ge-QD SHTs.

Figure 1 shows the schematic diagram, cross-sectional and plan-view transmission electron microscopy (TEM)/energy-dispersive X-ray spectroscopy (EDS) mapping, and scanning TEM (STEM) micrographs of studied Ge-QD SHTs. A ~ 20 nm Ge QD couples to boron-doped Si source/drain via tunneling barriers of 5 nm-thick SiO_2_/Si_3_N_4_ and to the top plunger-gate of poly-Si via 50 nm-thick gate oxide. Details for the fabrication of our Ge QD SHTs have been described elsewhere^17^.

**Figure 2:**
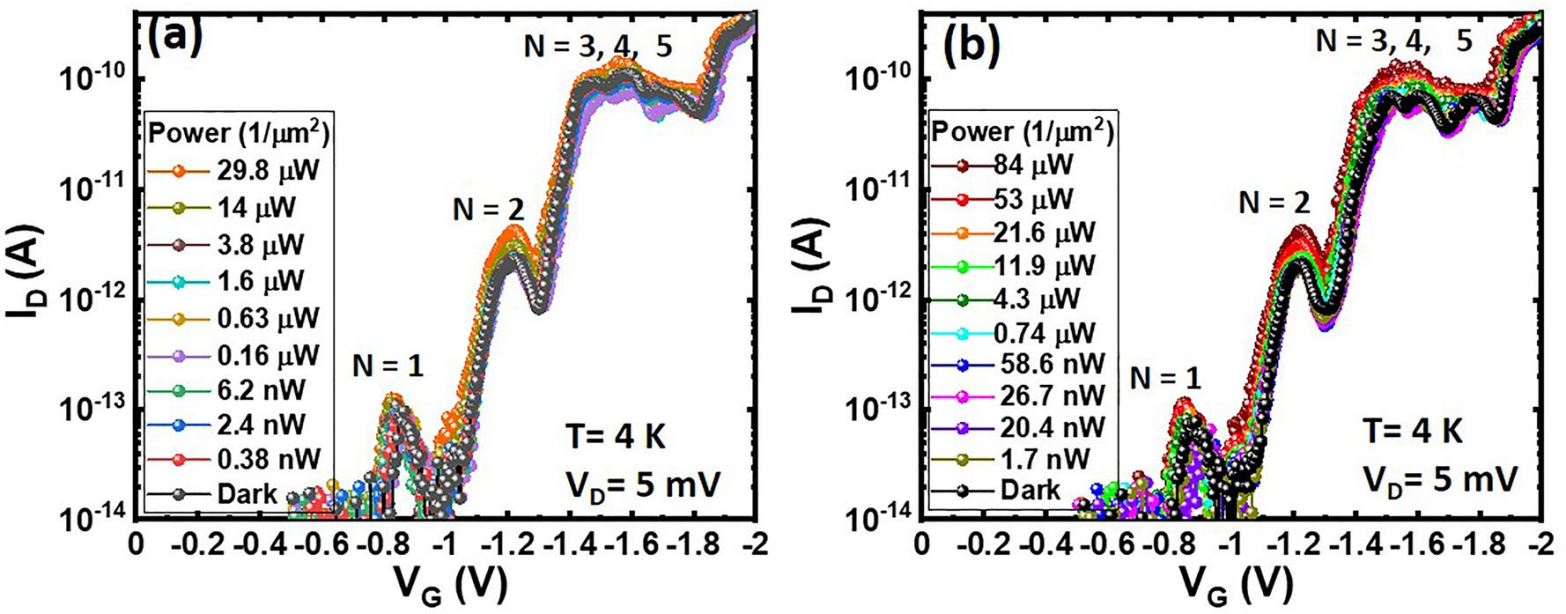
Power density-dependent I_D_–V_G_ characteristics of Ge-QD SHTs measured at V_D_ = 5 mV, T = 4 K and under illumination at λ = (**a**) 1310 nm and (**b**) 1550 nm and in the darkness.

Figure 2a, b show *I*_D_–*V*_G_ characteristics of Ge-QD SHTs measured in the darkness and under λ = 1310 nm/1550 nm illumination corresponding to photon energies of 0.8 eV/0.95 eV, which are smaller than the bandgap energy of 1.46 eV for a Ge QD with diameter of 20 nm^18–20^. It is clearly seen that in the darkness, the first tunneling current peak appears at V_G_ = − 0.82 V and is accompanied by a series of tunneling current peaks at − 1.23 V, − 1.49 V, − 1.6 V, and 1.78 V. The experimental observations of (1) invisible tunneling current peaks at V_G_ > − 0.8 V in combination with (2) irregular spacings between neighboring current peaks at V_G_ ranging from − 0.8 to − 2 V are a strong testament to our Ge QD SHTs operating in the few-hole regime. Tunneling current peaks located at − 0.82 V, − 1.23 V, − 1.49 V, − 1.6 V, and − 1.78 V correspond to the hole number of *N* = 1, 2, 3, 4, and 5, respectively. Illuminations at λ = 1310 nm or 1550 nm with irradiation power density as high as 10 µW/µm^2^ make the current peak, corresponding to the single-hole tunneling (*N* = 1) through the lowest energy level (*E*_*h*_), a slight shift toward positive V_G_ by ΔV_G_ ≈ 0.035 V, whereas the positions of the higher-order current peaks remain unchanged.

**Figure 3:**
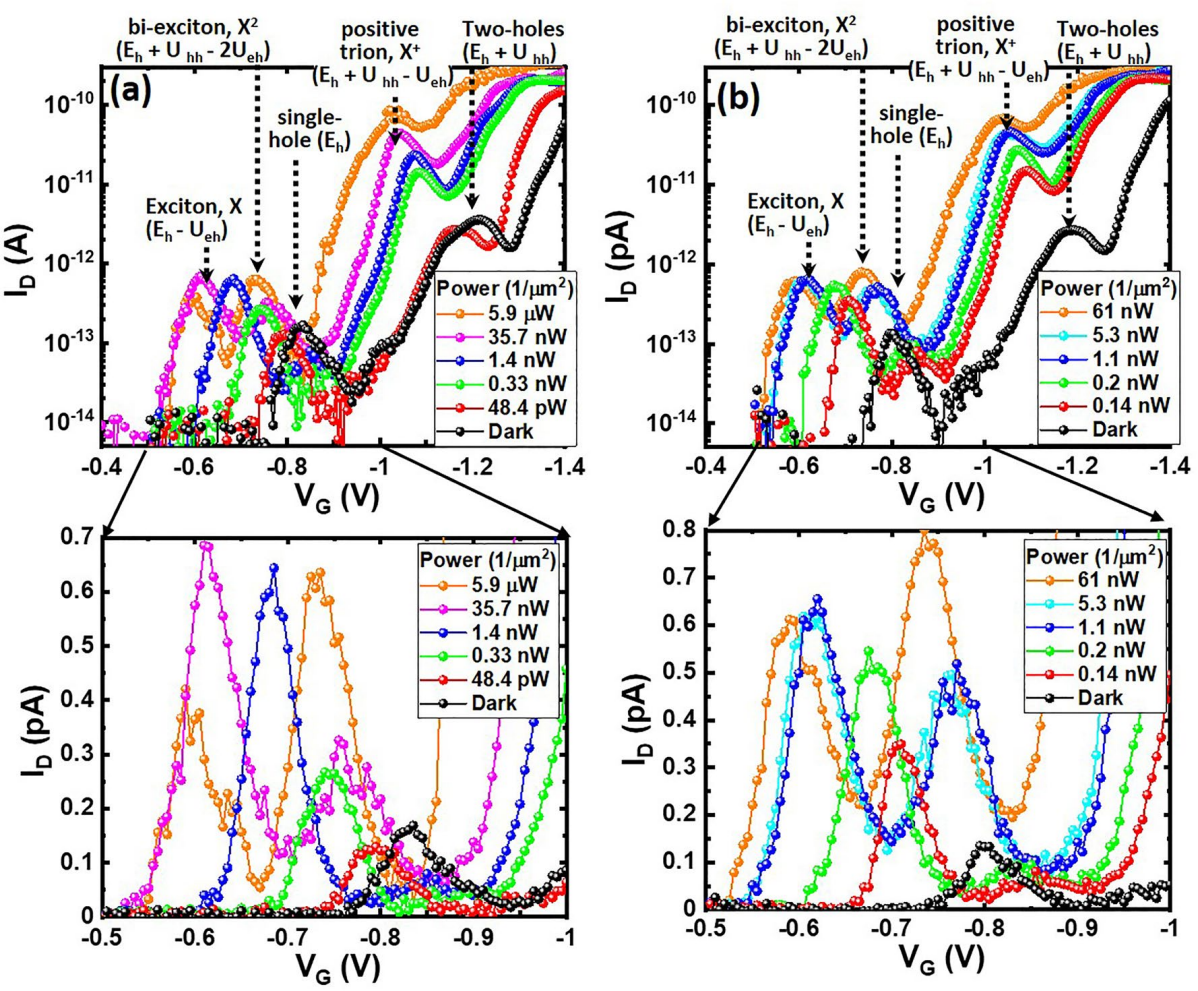
Power density-dependent I_D_-V_G_ characteristics of Ge-QD SHTs measured at V_D_ = 5 mV, T = 4 K under λ = (**a**) 850 nm and (**b**) 405 nm illumination and in the darkness. Insets are enlarged transfer curves showing the evolution of tunneling current peaks arising from states of single-hole, bi-exciton, and exciton with increasing illumination power.

Dramatic changes occur to the tunneling-current spectroscopy of Ge-QD SHTs when photon energy matches or is larger than the bandgap energy of the studied Ge QD (for instance, illumination at the wavelength of 405–850 nm corresponding to photon energy of 1.46–3.06 eV). The first important finding of notes from λ = 850 nm illumination is that an increase in the irradiation power density appears to make both the first (*N* = 1) and the second (*N* = 2) tunneling-current peaks systematic shifts toward positive V_G_ in combination with a considerable enhancement in current intensity (Fig. 3a). A detailed look at the photocurrent spectra at V_G_ = − 0.5 to − 1 V (as shown in the inset in Fig. 3a) reveals that when the optical power density increases to ~ 10 nW/µm^2^, the positive shift of these two current peaks saturates at V_G_ = − 0.6 V/− 1.01 V and an additional new current peak emerges at V_G_ = − 0.775 V. The third interesting observation is that the magnitude of the newly-generated photocurrent peak at V_G_ = − 0.775 V increases considerably and even becomes predominate when the power density reaches 5.9 µW/µm^2^. Figure 3b shows that illumination with photon energy of 3.06 eV (corresponding to the wavelength of 405 nm) induces similar photocurrent behaviors with the cases of λ = 850 nm illumination, including a positive V_G_ shift of the tunneling current peaks and the generation of new photocurrent peaks.

**Figure 4:**
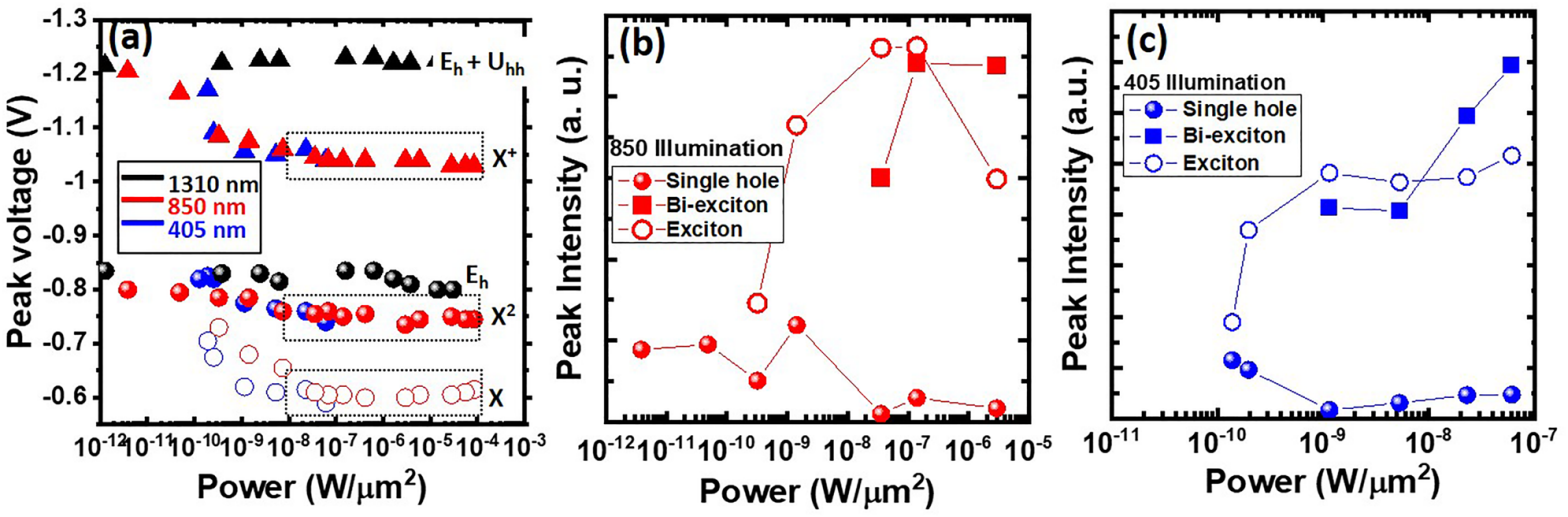
Power density-dependent (**a**) peak voltage of Ge-QD SHTs under λ = 405 nm, 850 nm, and 1310 nm illuminations. Power density-dependent peak intensity of tunneling current arising from single-hole, biexciton and exciton states under λ = (**b**) 850 nm and (**c**) 405 nm illumination.

Optical power density indeed influences the current-peak shift and new photocurrent-peak generation under λ = 405–1550 nm illuminations. Figure 4a clearly shows that λ = 1310 nm illumination (denoted by black symbols) makes no changes in the peak voltages (V_G_ = − 0.82 V and − 1.23 V) of tunneling current arising from single-hole and two-hole states, whereas a considerable positive shift in the peak voltage from − 0.82 to − 0.6 V and − 1.23 to − 1.01 V as well as the generation of new additional current peak at − 0.775 V are induced by illuminations at λ = 405 nm (denoted by blue symbols) and λ = 850 nm (red symbols). Notably, λ = 405 nm illumination makes the peak shifts saturated and new photocurrent peaks generated at much lower optical power densities (1.1 nW/µm^2^) than λ = 850 nm illumination does at 35.7 nW/µm^2^. The newly-generated current peak at − 0.775 V predominates in magnitude over the peak at − 0.6 V when irradiated at λ = 850 nm and λ = 405 nm with the optical power density larger than 0.19 µW/µm^2^ and 8.91 nW/µm^2^, respectively, as seen in Fig. 4b, c.

## Discussion

Our previous reports^18–20^ have experimentally demonstrated the controlled tunability of photoluminescence (PL) peak wavelength (energy) ranging from 350 to  1550 nm (0.8− 3.55 eV) by adjusting the Ge-QD diameter (*D*_QD_) from 3 to 90 nm. A strong testament to quantum size effects on our studied Ge QDs is manifested by a considerable blue-shift in PL peak energy (*E*_PL_) when the Ge QD diameter is smaller than 30 nm. The size-dependent PL peak energy of Ge QDs could be described using *E*_PL_ = 0.79 (eV) + 310/(*D*_QD_ (nm))^2^^18,20^.

**Figure 5:**
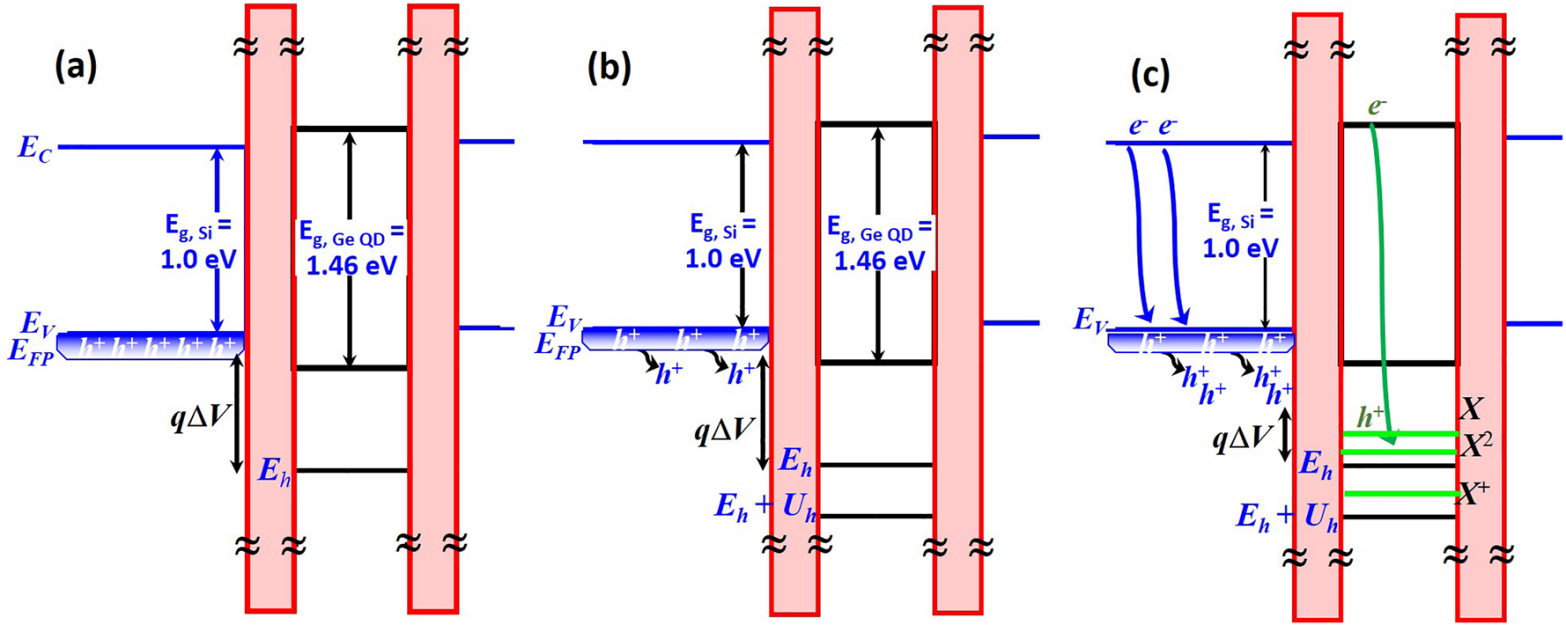
Energy band diagram of doped-Si reservoir/SiO_2_/Ge-QD/SiO_2_/doped-Si reservoir (**a**) in the darkness and under illumination at (**b**) 1310–1550 nm, (c) 405–850 nm.

It is seen in Fig. 2a, b that illumination at λ = 1310 nm/1550 nm is insufficient to excite electron–hole (*e*^–^–*h*^+^) pairs within the studied 20 nm Ge QD since photon energies of 0.95 eV/0.8 eV are smaller than its optical bandgap energy of 1.46 eV. Thereby, tunneling current spectroscopy of Ge-QD SHTs remains intact even when the excitation power density of λ = 1310–1550 nm irradiation is as high as 10 µW/µm^2^. A slight shift of the first current peak towards positive voltage (ΔV_G_ ~ 0.03 V) under high power density (10 µW/µm^2^) irradiation possibly originates from boson-assisted tunneling (BAT) effects^21–24^. BAT (including photon and other phonon modes indirectly excited by optical pumping) effects assume that conducting holes within the valence band of boron-doped Si source reservoir are excited to the lowest energy level (*E*_*h*_) by bosons (Fig. 5b), facilitating the onset of single-hole tunneling (*N* = 1) due to a reduced energy difference (*q*Δ*V*) between the lowest energy level (or the ground state, *E*_*h*_) of the QD and the chemical potential (or Fermi energy, *E*_*FP*_, _source_) of source reservoir as compared to the case in the darkness (Fig. 5a).

On the contrary, λ = 405–850 nm illuminations with photon energy of 1.46–3.06 eV allow photoexcitation of electron–hole pairs within a 20 nm Ge QD (Fig. 5c). The appearance of new photocurrent peaks at more positive V_G_ with respect to tunneling current peaks arising from single-hole (*N* = 1) and two-hole (*N* = 2) states is a strong testament to the photoelectron storage within the Ge QD, suggesting that the generation rate of photocarriers is higher than the tunneling rate of holes through Ge QD/Si_3_N_4_ system in our Ge-QD SHTs. The experimentally measured magnitude (sub-pA) of tunneling current in Figs. 2 and 3 suggests the time for hole tunneling through the Ge QD/Si_3_N_4_ system is approximate sub-µs, which is much longer than the generation time of sub-ns for photoelectron-hole pairs within Ge QDs from our transient photoluminescence measurement^19^.

The coexistence of tunneling hole and photoelectron-hole pairs results in the renormalization of energy levels and even the creation of new transport levels of the Ge QD. This is because photogenerated holes in the Ge-QD induce the repulsive, intralevel Coulomb interactions (*U*_*hh*_) with the tunneling holes causing the Coulomb blockade, whereas the attractive, interlevel Coulomb interaction (*U*_*eh*_) between the photoelectrons and tunneling holes gives rise to the binding of excitons^12^. The strengths of these Coulomb interactions are inversely proportional to the QD size. In general, *U*_*hh*_ is larger than *U*_*eh*_ and the difference between *U*_*hh*_ and *U*_*eh*_ becomes large in small QDs^25^. Therefore, SHTs with small QDs are desirable to have a distinguishable difference between *U*_*hh*_ and *U*_*eh*_ so as to resolve exciton binding-energy from well-separated photocurrent peaks.

Low-to-medium-level optical pumping generated a small number of photoelectrons and photoholes within the Ge QDs. The coexistence of tunneling holes and photogenerated electron–hole pairs forms the exciton complexes, creating new transport energy levels characterized by the exciton (*E*_*h*_ − *U*_*eh*_) and biexciton (*E*_*h*_ + *U*_*hh*_ − 2*U*_*eh*_) below the original ground state (*E*_*h*_) corresponding to single-hole tunneling in the darkness as shown in Fig. 3^12^. Besides, additional energy level due to the positive trion (*E*_*h*_ + *U*_*hh*_ − *U*_*eh*_) is photo-created between the single-hole state (*E*_*h*_) and two-hole state (*E*_*h*_ + *U*_*hh*_). It is seen from Fig. 3 that the current peak originating from the transport level of the negative trion (*E*_*h*_ − 2*U*_*eh*_) is not observable at V_G_ > − 0.6 V, possibly due to the charge transport being blocked by the Fermi sea of source reservoirs. One important finding of notes from Fig. 3 is that new current peaks corresponding to the exciton state (*X*), biexciton state (*X*^2^), and positive trion state (*X*^+^) are photogenerated at V_G_ = − 0.6 V, − 0.775 V, and 1.01 V, respectively, in addition to the single-hole tunneling through the ground state (*E*_*h*_) at V_G_ = − 0.82 V and two-hole tunneling through the hole-hole charging state (*E*_*h*_ + *U*_*hh*_) at V_G_ = − 1.23 V. These well-resolved photocurrent peaks allow to extract the exciton binding energy (*U*_*eh*_) and hole-hole charging energy (*U*_*hh*_) from the corresponding gate-voltage spacings (ΔV_G_) of V_G, single-hole state_—V_G, *X*_ = 0.22 V and V_G, two-hole state_—V_G, single-hole state_ = 0.41 V, respectively. Gate modulation factor (α) of ~ 0.122 was extracted from the slopes of Coulomb diamonds in the Coulomb stability diagram of Ge QD SHTs (not shown here)^17^. Estimated values of *U*_*hh*_ and *U*_*eh*_ are 50 meV and 27 meV, respectively, using *U* = αΔ*V*_G_. The experimentally-extracted values of *U*_*hh*_ and *U*_*eh*_ also explain well the peak-voltage shifts arising from bi-exciton state (*X*^2^) and positive trion state (*X*^+^) shown in Fig. 3.

We have also performed theoretical calculations on the Coulomb interactions between particles, including hole-hole (*U*_*hh*_), electron–hole (*U*_*eh*_), and electron–electron (*U*_*ee*_) for a Ge QD embedded within SiO_2_. These calculations were based on the effective mass method, considering a finite potential barrier height of 3.1 eV and 5.1 eV for electrons and holes, respectively, at the interface boundary between the Ge QD and SiO_2_. For a Ge QD with diameter of 20 nm, we derived the following values for the particle Coulomb interactions: *U*_*hh*_ = 18.0 meV and *U*_*eh*_ = 16.0 meV based on our calculations using effective masses of 0.12*m*_0_ and 0.284*m*_0_ for electrons and holes in the Ge QD, respectively. Our calculated trend of *U*_*hh*_ > *U*_*eh*_ aligns with the experimental estimation derived from photocurrent spectroscopy of Ge QD SHTs. However, the magnitude of calculated *U*_*hh*_ and *U*_*eh*_ appears to be smaller than that of experimentally-extracted data. Our calculation possibly underestimated the actual Coulomb interactions between particles. This is because that in our calculation, the image charge effect resulting from a significantly large difference in the dielectric constants between Ge and SiO_2_ as well as the screen-potential effect between particles were not considered. Both effects can potentially enhance particle Coulomb interactions and increase the energy difference between *U*_*hh*_ and *U*_*eh*_^26^.

In addition to generating photocarriers within the Ge QD, illumination at λ = 405 nm–850 nm potentially creates electron–hole pairs within boron-doped Si reservoirs and increases the number of conducting holes occupying higher states in the valence band. Consequently, the optical-pumping process facilitates hole tunneling by reducing the energy difference between the lowest energy level of the Ge QD and the chemical potential of Si source reservoir (Fig. 5c). Thereby, increasing optical power density enhances shifts in the tunneling-current peaks. It is important to note that the increase in the chemical potential of Si reservoirs by increasing optical power density eventually saturates when the generation rate of electron–hole pairs equals to the recombination rate. Therefore, the shift in the tunneling current peaks is only observable at low optical-power density conditions. In fact, Reference 12 did not consider the change in the chemical potential of reservoirs with respect to optical pumping powers so that the calculated peak positions are constant and independent of optical pumping powers.

Another important finding of notes from Fig. 4b, c is that concurrent with the formation of exciton and biexciton complexes within the Ge QD, the current peak of single-hole state appears to be suppressed. In particular, we observe a considerable decline in the current intensity of single-hole state once the biexciton current peak starts to emerge at optical power density of 36 nW/µm^2^ and 1.15 nW/µm^2^ under illumination at λ = 850 nm and 405 nm, respectively. Notably, the current intensity of the exciton configuration exceeds that of the biexciton configuration when illuminated at λ = 850 nm and 405 nm with optical power densities < 0.19 µW/µm^2^ and < 8.91 nW/µm^2^, respectively, beyond which the cross-over occurs and the biexciton current peak surpasses the exciton peak in magnitude. The current intensity of either exciton or biexciton states, as illustrated in Fig. 4b, c, is influenced by the likelihood of such complexes formation, which essentially relies on the occupation numbers of electron and hole^12^. Consequently, the current peak arising from biexciton complex (comprising two electrons and two holes) is prone to emerge under strong optical pumping conditions. The observed behaviors of tunneling-current intensity for exciton and biexciton states in response to optical pumping power density are akin to the power-dependent emission spectra of exciton and biexcition states in an InGaAs QD single photon generator^27^.

## Conclusion

We have investigated photoexcitation effects on tunneling-current spectroscopy of Ge-QD SHTs operating in the few-hole regime. The devices were illuminated at λ = 405–1550 nm with excitation power density varying from 10 to 10 µW/µm^2^. Our study focused on a small Ge QD with diameter of 20 nm, which exhibits significant energy-level separations and a large hole-hole charging energy. The notable disparity between the exciton-complex states and hole-hole charging energy allows the identification of corresponding photocurrent peaks. Consequently, we were able to directly determine the hole-hole charging energy and exciton binding energy through photocurrent spectroscopy of Ge-QD SHTs. This approach offers a unique advantage over conventional techniques such as photoluminescence or electrically driven emission spectrum measurements.

## Methods

### The fabrication of Ge-QD SHTs

The fabrication started with an SOI substrate with a 50 nm-thick, boron-doped Si (100) layer. A triangle-shaped Si trench (denoted as Trench I) was produced using electron-beam lithography (EBL) and SF_6_/C_4_F_8_ plasma etching. Next, bi-layers of 10 nm-thick Si_3_N_4_ and 25 nm-thick polySi_0.85_Ge_0.15_ were sequentially deposited using low-pressure chemical vapor deposition (LPCVD) for conformal encapsulation over Trench I. Following a direct etch back, spacer layers of poly-Si_0.85_Ge_0.15_ with width/height of 25 nm/30 nm were produced at the sidewalls of Si_3_N_4_-encapsulated Trench I. The length of the poly-Si_0.85_Ge_0.15_ spacer islands at the included-angle location of Trench I in combination with Trenches II and III (forming Si electrodes for gate, source, and drain (G/S/D)) were simultaneously delineated using EBL and plasma etching processes. Subsequently, thermal oxidation at 900 °C in an H_2_O ambient converted the poly Si_0.85_Ge_0.15_ spacer island to a single Ge QD at the corner of Trench I. Concurrent with the Ge QD formation, the connection between three Si electrodes for G/S/D was also converted to SiO_2_ since the sidewalls of Si Trenches II/III are subjected to thermal oxidation as well. Therefore, the thermally-grown SiO_2_ layers electrically isolate each of the G/S/D electrodes. Finally, contact and metallization processes completed the device fabrication^17^.

### Optical characterization

All electrical and optical characterizations were performed in vacuum. λ = 405 nm–1550 nm illuminations with spot sizes of 10 × 10 µm^2^ were incident to the Ge-QD SHTs through a lens fiber with an angle of 80 degrees from the horizon. Current–voltage characteristics of Ge QD-SHTs were measured within a Lakeshore CRX-4K closed-cycle liquid helium refrigerator-cooled vacuum-sealed probe station using an Agilent B1500 semiconductor device analyzer equipped with a B1517A high-resolution source monitor unit/auto sense and switch unit (the current measurement resolution is in femtoampere range (< 10 fA)) both in darkness and under λ = 405–1550 nm illuminations.

### Supplementary Information

Supplementary Information.

## Data Availability

The datasets used and/or analyzed during the current study available from the corresponding author on reasonable request.
