# Supplementary material for: Determination of exciton binding energy using photocurrent spectroscopy of Ge quantum-dot single-hole transistors under CW pumping
Source: Sci Rep. 2023 Aug 31;13:14333. doi: 10.1038/s41598-023-41582-8 (PMC10471612; doi:10.1038/s41598-023-41582-8)
Supplement: Supplementary file 1 — Supplementary Information. [file 41598_2023_41582_MOESM1_ESM.pdf]

Received 11 July 2022; revised 14 October 2022 and 11 December 2022; accepted 28 December 2022. Date of publication 9 January 2023; date of current version 21 February 2023. The review of this article was arranged by Editor N. Collaert.

*Digital Object Identifier 10.1109/JEDS.2023.3235386*

# Germanium Spherical Quantum-Dot Single-Hole Transistors With Self-Organized Tunnel Barriers and Self-Aligned Electrodes

**CHI-CHENG LAI, R. C. PAN, I-HSIANG WANG (Student Member, IEEE), T. GEORGE, HORNG-CHIH LIN (Senior Member, IEEE), AND PEI-WEN LI (Senior Member, IEEE)**

Institute of Electronics, National Yang Ming Chiao Tung University, Hsinchu 30010, Taiwan

CORRESPONDING AUTHOR: P.-W. LI (e-mail: pwli@nycu.edu.tw)

This work was supported in part by the National Science and Technology Council under Grant NSTC 111-2119-M-A49-003 and Grant 109-2221-E-009-022-MY3, and in part by the Higher Education Sprout Project of National Yang Ming Chiao Tung University and Ministry of Education, Taiwan.

**ABSTRACT** We report the fabrication and electrical characterization of single-hole transistors (SHTs), in which a Ge spherical quantum dot (QD) weakly couples to self-aligned electrodes via self-organized tunnel barriers of $Si_3N_4$. A combination of lithographic patterning, sidewall spacers, and self-assembled growth was used for fabrication. The core experimental approach is based on the selective oxidation of poly-SiGe spacer islands located at the specially designed included-angle locations of $Si_3N_4$/Si-trenches. By adjusting processing times for conformal deposition, etch back and thermal oxidation, good tunability in the Ge QD size and its tunnel-barrier widths were controllably achieved. Each Ge QD is electrically addressable via self-aligned Si gate and reservoirs, thus offering an effective building block for implementing single-charge devices.

**INDEX TERMS** Ge, quantum dot, single-hole transistors.

## I. INTRODUCTION

Single-electron or -hole transistors (SETs or SHTs) offer great promise in a vast landscape of applications for ultra-fine sensing, precision metrology, and in particular, readouts for quantum registers. Charge transport within SETs is controlled at single charge precision based on Coulomb blockade effects, [1] featuring current oscillation and plateaus under gate and drain modulation. The core structure of a semiconductor quantum-dot (QD) SET is similar to that of a MOSFET, except that the continuous conducting channel between source and drain (S/D) reservoirs for a MOSFET is replaced by a single QD capacitively coupled to S/D through tunnel barriers for a SET. While the device structure of SETs is straightforward, the fabrication of controllable sizes and widths for the QDs and their tunnel barriers, respectively, at nanometer-scales for Si SETs has proven challenging. References [2], [3], [4], [5], [6] This is because Si QDs with diameters smaller than 5 nm (as dictated by the Bohr radius of 4.9 nm for Si [7]) are desired to have well-separated energy levels and thereby be immune to thermal noise above cryogenic temperatures. Additionally, good control over the thickness, potential height, and interfacial properties of the intimately coupled tunnel barriers is essential to achieve measurable tunneling current (> pA) with large peak-to-valley current ratio (PVCR) or signal-to-noise ratio (SNR) for subsequent signal processing. Of most concern is that multiple electrodes in close proximity to a nm-scale QD will result in significant inter-electrode crosstalk [8] and parasitic capacitances. All the above-mentioned nanofabrication-related low PVCR-related effects not only result in driving down the operational temperature of Si-QD SETs to milli-Kelvin levels, but also impact the fabrication reproducibility and operational reliability of Si-QD SETs.

In contrast, the large Bohr radius of 24.9 nm in Ge [7] allows easier fabrication of Ge-QD SETs and SHTs. We have already reported CMOS-compatible fabrication approaches

This work is licensed under a Creative Commons Attribution 4.0 License. For more information, see https://creativecommons.org/licenses/by/4.0/

for the controllable, self-organized growth of spherical Ge QDs/$SiO_2$ shells within $Si_3N_4$ and Si at designated spatial locations. References [9], [10], [11], [12], [13] Most importantly, our Ge QDs come with their inherent confinement barriers of thermally-grown $SiO_2$ shells within the embedding $Si_3N_4$ layers in a self-organized manner, thereby offering an effective building-block for the fabrication of Ge-QD SETs and SHTs [14], [15], [16].

In this paper, we advance the fabrication of Ge-QD SHTs with both self-organized tunnel barriers of $Si_3N_4$ and self-aligned electrodes of Si. Tunneling current of the Ge-QD SHTs measured at T = 4-40 K is also reported.

## II. EXPERIMENTAL FABRICATION OF GE QDS SHTS

The fabrication of Ge-QD SHTs with self-organized tunnel barriers of $SiO_2/Si_3N_4$ and self-aligned heavily-doped Si electrodes is described in Fig. 1. Reference [14] Starting with an SOI substrate with a 50 nm-thick, boron-doped Si (100) layer, a triangle-shaped Si trench (denoted as **Trench I)** was produced using electron-beam lithography (EBL) and $SF_6/C_4F_8$ plasma etching (Fig. 1a). Next, bi-layers of 10 nm-thick $Si_3N_4$ and 25 nm-thick poly-$Si_{0.85}Ge_{0.15}$ were sequentially deposited using low-pressure chemical vapor deposition (LPCVD) for conformal encapsulation over the **Trench I** (Fig. 1b). Following a direct etch-back process (Fig. 1c), spacer layers of poly-$Si_{0.85}Ge_{0.15}$ with width/height of 25 nm/30 nm were produced at the sidewalls of $Si_3N_4$-encapsulated **Trench I**. The length of the poly-$Si_{0.85}Ge_{0.15}$ spacer islands at the included-angle location of **Trench I** in combination with **Trenches II** and **III** (forming Si electrodes for gate, source, and drain (G/S/D)) were simultaneously delineated using EBL and plasma etching processes (Fig. 1d). Subsequently, thermal oxidation at 900°C in an $H_2O$ ambient converted the poly-$Si_{0.85}Ge_{0.15}$ spacer island to a single Ge QD at the corner of **Trench I** (Fig. 1e). Concurrent with the Ge QD formation, the connection between three Si electrodes for G/S/D was also converted to $SiO_2$ since the sidewalls of Si **Trenches II/III** are subjected to thermal oxidation as well. Therefore, the thermally-grown $SiO_2$ layers electrically isolate each of the G/S/D electrodes. Finally, contact and metallization processes completed the device fabrication (Fig. 1f).

Current-voltage characteristics of Ge QD-SHTs were measured within a Lakeshore CPX-VF liquid-nitrogen cooled vacuum-sealed probe station using an Agilent B1500 semiconductor device analyzer equipped with a B1517A high-resolution source monitor unit/auto sense and switch unit (the current measurement resolution is in femtoampere range (< 5 fA)).

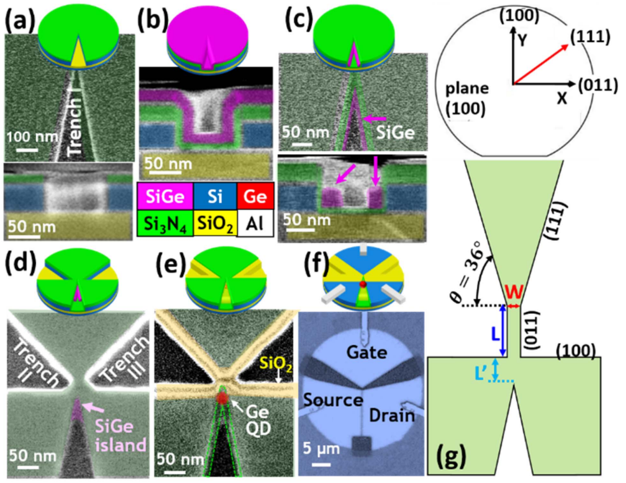


**FIGURE 1. Schematic diagrams and corresponding SEM micrographs showing the precision fabrication of Ge QD SHTs at desired locations. (a) Lithographically-patterned Si Trench I. (b) Deposition of $Si_3N_4$ and poly-$Si_{0.85}Ge_{0.15}$ layers. (c) Formation of poly-$Si_{0.85}Ge_{0.15}$ spacer layers at Trench I sidewalls. (d) Patterning of Trenches II/III and a poly-$Si_{0.85}Ge_{0.15}$ spacer island at the included-angle location of Trench I. (e) Formation of a single Ge QD with self-aligned G/S/D electrodes by thermal oxidation. (f) Contact and metallization for forming electrodes. (g) Layout design of Si connection among G/S/D electrodes.**

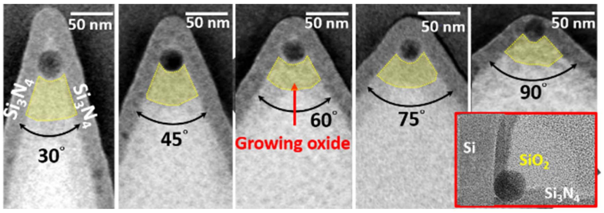


**FIGURE 2. Plan-view TEM micrographs shows the dependence of $Si_3N_4$ layer penetration depth by Ge QDs as a function of the included-angle of the Si trenches following thermal oxidation at 900 °C. Inset is the cross-sectional TEM micrograph showing the Ge QD is conformally capped with thermally-grown $SiO_2$ that is generated in a self-organized manner via the thermal oxidation of the Si content of the former poly-SiGe spacer island.**

## III. RESULTS

### *A. PRECISION FABRICATION OF SELF-ASSEMBLED GE-QD AT THE DESIGNATED INCLUDED-ANGLE LOCATION OF SI TRENCHES*

Plan-view transmission-electron microscopy (TEM) observations show that following thermal oxidation for 40 min, a single Ge QD is produced precisely at the included-angle location of the **Trench I** with conformal overlayers of $Si_3N_4$ (Fig. 2). Reference [14] The Ge QD is formed in a self-assembled approach via Ge interstitial condensation, Ostwald ripening, and the ultimate complete coalescence of Ge nanocrystallites generated from the original poly-$Si_{0.85}Ge_{0.15}$ island during the selective oxidation process. Reference [9] A significant fabrication advantage of our approach is the size tunability of the Ge QDs that is essentially determined by the total Ge content of the poly-$Si_{1-x}Ge_x$ spacer island [9], [10], [11], [12]. The Ge QD size is, by definition, smaller than the geometric size of the lithographically-patterned poly-$Si_{1-x}Ge_x$ spacer island. For instance, thermal oxidation of a poly-$Si_{0.85}Ge_{0.15}$ spacer island with width/height/length of 20 nm/35 nm/35 nm produces a 20 nm-diameter Ge QD, thus eliminating the need for high-resolution EBL for the fabrication of tiny QDs.

### B. CONTROLLABLE GE QD PENETRATION INTO THE $Si_3N_4$ LAYER

Figure 2 shows highly symmetrical spacer layers of $Si_3N_4$ being produced at the sidewalls of **Trench I** thanks to the conformal deposition using LPCVD. It is important to note that the overlayer of $Si_3N_4$ at the sidewalls of **Trench I** is specially designed to serve as the tunnel barriers between the Ge QD and Si reservoirs as well.

The sidewall spacer layer of $Si_3N_4$ is indeed responsible for the controllable placement of Ge QDs via a unique Ge QD migration within the already-formed $SiO_2$ layers and through the spacer layer of $Si_3N_4$ in the solid state during the thermal oxidation process. Our extensive experimental observations [9], [12] have elucidated the fundamental mechanisms responsible for this unique Ge QD migration via a combination of symbiotic interactions of Si, Ge, and O interstitials occurring simultaneously. We discovered that the Ge QD is able to catalyze the release of Si interstitials through local oxidation of the proximal $Si_3N_4$. Reference [12] The released Si interstitials in turn promote the Ge QD migration through its surrounding $SiO_2$ matrix along the Si interstitial concentration gradient towards the $Si_3N_4$ layer via a dynamic $SiO_2$ destruction/construction mechanism near the migrating Ge QD surface. Reference [9] In brief, during the thermal oxidation process, Si interstitials catalyze the decomposition of the $SiO_2$ ahead of the migrating Ge QD and facilitate the subsequent re-formation of $SiO_2$ in its wake.

It is clearly seen in Fig. 2 that the forehead of the Ge QD penetrates the sidewall spacer layer of $Si_3N_4$. Thereby, our approach indeed provides yet another vital fabrication advantage for tuning the tunnel-barrier widths via the controllable penetration of the $Si_3N_4$ layer by the Ge QD. Fig. 2 shows that for a given process time of 40 min for thermal oxidation, an increase in the included angle (from 30° to 90°) of **Trench I** through layout design and nanofabrication results in the enhanced penetration of $Si_3N_4$ spacer layers by the Ge QD, further reducing the tunnel-barrier width between the Ge QD and Si electrodes [14].

An important finding of note is that behind the migrating Ge QD, a 25–30 nm-thick, thermally grown $SiO_2$ layer (as highlighted by the yellow boxes in Fig. 2) simultaneously and conformally cap the resulting Ge QD. The capping layer of $SiO_2$ over the Ge QD is formed in a self-organized manner via the generation from the Si content of the former poly-SiGe spacer island by the selective oxidation process, providing good passivation for the Ge QD.

### C. SELF-ALIGNED PLUNGER GATE

Isolating and modulating only the QD potentials for a tiny QD located among multiple electrodes (G/S/D) by a plunger gate is very challenging. Therefore, it is highly desirable that the plunger gate is precisely self-aligned to the QD with a minimum of overlap with the S/D electrodes. In our fabrication approach, Si **Trenches II/III** and a poly-SiGe spacer island within **Trench I** were simultaneously generated using a single-step lithographic-patterning process (Fig. 1d). The designed length (L)/width (W) of the Si connection between Trench II and Trench III as well as the separation (L') between Trench II/III and Trench I are 30 nm/40 nm and 40 nm, respectively (Fig. 1g). It is important to note that the overlayer of $Si_3N_4$ for **Trench I** prevent the sidewalls of Trench I from being oxidized (Fig. 1b), whereas the exposed (100) and (110) sidewalls of Si Trenches II and III are directly subjected to oxidation (Fig. 1g). According to the growth kinetics of Si oxidation at 900 °C in an $H_2O$ ambient, 40 min thermal oxidation grows 100 nm-thick and 140 nm-thick $SiO_2$ layers by consuming 45 nm-thick (100) Si and 63 nm-thick (110) Si, respectively. Therefore, concurrent with the formation of Ge QD by using the thermal oxidation at 900 °C for 40 min, the connection of Si among Trenches I/II/III is completely disconnected since all exposed (110) and (100) sidewalls of Si connection (perpendicular to and along the direction of A–A', respectively,) are subjected to thermal oxidation. In this way, electrodes of G/S/D were separately formed and self-aligned to each other automatically since the growing oxide layer serves to electrically isolate each electrode from the other. Also, with our fabrication approach, there is negligible overlap between the plunger gate and the S/D electrodes so as to effectively eliminate inter-electrode cross-talk.

### D. SELF-ALIGNED SI SOURCE/DRAIN RESERVOIRS

Cross-sectional electron dispersive spectroscopy (EDS) mapping (Fig. 3) observations clearly show that at the included-angle location of Trench I, a single Ge QD couples to S/D reservoirs via nearly identical 9 nm-thick sidewall spacers of $Si_3N_4$ thanks to a conformal deposition of $Si_3N_4$ over the trench. It is a known fact that $Si_3N_4$ has a much lower oxidation rate than that for both SiGe and Si. Thereby, the $Si_3N_4$ overlayer for **Trench I** indeed is an effective oxidation mask, protecting the Si S/D from oxidation attack during the subsequent thermal oxidation process for forming the Ge QD. In this way, the widths of tunnel barriers between the Ge QD and Si reservoirs are essentially determined by the thicknesses of the $Si_3N_4$ overlayers at the sidewalls of **Trench I**. Therefore, we are able to tailor the tunnel-barrier widths with nm-scale precision by adjusting the deposition time of LPCVD-$Si_3N_4$ layers.

### E. ELECTRICAL CHARACTERISTICS OF GE SHTS AT 4 – 40 K

Our Ge-QD SHTs show progressive Coulomb staircase behavior under drain ($V_D$) modulation at T = 4 K and 20 K. Fig. 4(a) shows that at T = 4 K and $V_G = -3.5$ V, current-plateaus appear when $V_D$ goes beyond Coulomb-gap voltages of $V_D+ > 0.65$ V or $V_D- < -0.45$ V. An increase in the magnitude of $V_G$ from 0 V to −3.5 V not only reduces Coulomb-gap voltages by facilitating the line-up of the Ge-QD energy levels with the Fermi energy of source reservoir, and also makes these current plateaus more prominent.

The first current plateau at $V_D \cong +0.70$ V appears to have negative differential conductance (NDC), that is, $G_D$

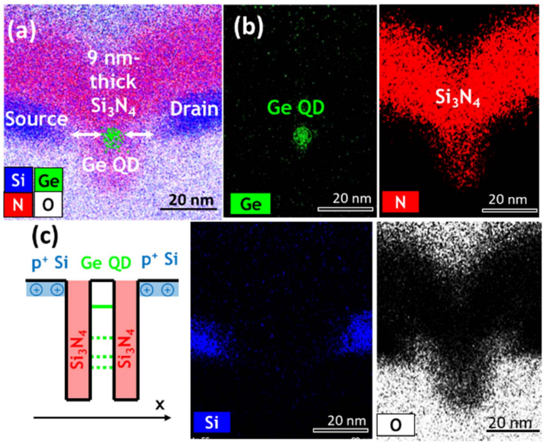


**FIGURE 3.** **(a, b) Cross-sectional EDS maps of the distributions of oxygen, germanium, nitrogen, and silicon atoms generated during the formation of a Ge QD at the included-angle location of a $Si_3N_4$/Si trench. (c) Hole quantum confinement energy levels within the Ge QD generated by the $Si_3N_4$/Si layers.**

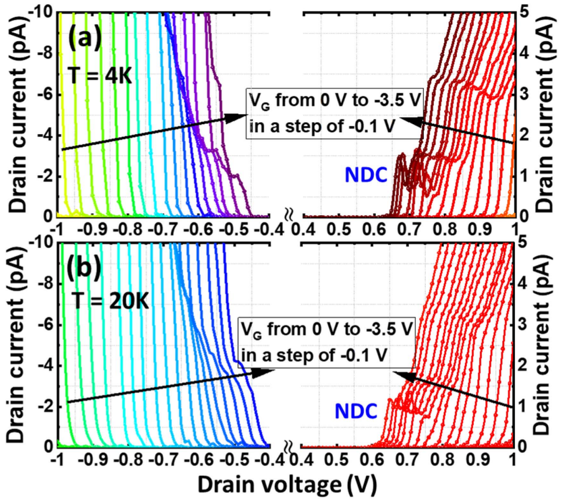


**FIGURE 4.** **$I_D$-$V_D$ characteristics of Ge-QD SHTs measured at T = (a) 4 K and (b) 20 K.**

$\equiv \partial I_D/\partial V_D < 0$, whereas staircase-like current behavior ($G_D \geq 0$) is observable for the first current plateau at $V_D \cong -0.5$ V. Such drain polarity-dependent Coulomb gaps and tunneling-current behaviors suggest a slight difference in the tunneling rates between the Ge QD/Si-source and Ge QD/Si-drain electrodes. The asymmetrical tunneling rates lead to shell-tunneling (holes tunnel through bare energy levels with no inter-charge Coulomb interaction within a QD. That is, no charge accumulation within the QD because the tunneling rate for holes injecting into a QD from source reservoir is smaller than that for holes leaving for drain.) and shell-filling (holes tunnel out of a small QD much more slowly than they can be fed in) processes for $V_D > 0$ and $V_D < 0$ cases, respectively. The NDC features at $V_D = 0.70$ V and current-staircase at $V_D = -0.5$ V are still observable at T = 20 K (Fig. 4(b)).

Figure 5 show measured gate-induced oscillatory tunneling currents at $V_D = 0.1$ V – 0.45 V and T = 4 K – 40 K. It is clearly seen in Fig. 5(a) that at small $V_D = -0.2$ V and T = 4 K, two distinct current peaks are present at $V_G = -4.6$ V and $-6.05$ V, respectively. When $V_D$ is increased from $-0.2$ V to $-0.35$ V, there appear to be more oscillatory current peaks accompanied by the shift of current peaks toward smaller $V_G$. This is because increasing $V_D$ facilitates more energy levels of the Ge QD approaching the Fermi energy of source reservoir, creating more transmission resonance conditions for hole tunneling. On the other hand, increasing temperature makes the oscillatory current peaks broaden and even merge together since the higher thermal noise washes out discrete energy levels. It is important to note that at $V_D = -0.2$ V, the oscillatory current peaks at $V_G = -4.6$ V and $-6.05$ V are nearly invariant with temperature and the current-valley or background value of $\sim$10 fA is very close to the $\sim$ 5 fA resolution for our characterization system. Very low current valleys are strong evidence for a precisely self-aligned plunger gate that suppresses gate-induced tunnel-barrier lowering by minimizing the gate overlap with the S/D electrodes.

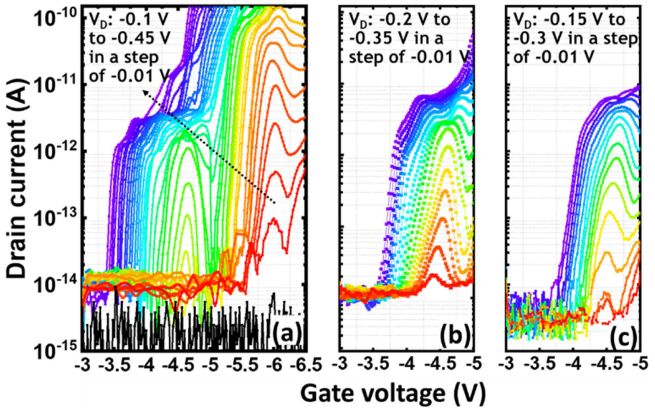


**FIGURE 5.** **$I_D$-$V_G$ characteristics of Ge-QD SHTs measured at T = (a) 4 K, (b) 20 K, and (c) 40 K.**

The contour plot of differential conductance as a function of $V_G$ and $V_D$ is shown in Fig. 6. Sharp boundaries of the Coulomb diamond allowed us to extract the gate modulation factor ($\alpha \equiv C_G/(C_D + C_S + C_G)$) derived from the slopes of the diamond. Extracted capacitance ratios of $C_D : C_S : C_G = 4.0 : 4.7 : 1$ suggest an $\alpha = 0.105$ and total capacitance of 0.11 aF, with estimated single-hole addition energies for $N = 0 \rightarrow 1$ and $1 \rightarrow 2$ being 145 meV and 49 meV, respectively, estimated using $E_a = \alpha \Delta V_G$.

## IV. DISCUSSION

Our experimentally observed aperiodic Coulomb oscillations in combination with NDC characteristics are strong testaments to large, nonuniform quantum-level spacings ($\Delta$E) in our Ge QD caused by quantum confinement effects. Similar experimental observations have also been reported in Ge QD SHTs [15], [16] and Si-SETs [17], [18], [19], [20]. It is a known fact that for a SET/SHT, the addition energy ($E_{add}$) required for injecting an additional charge into the QD comprises the charging energy ($E_C$) arising

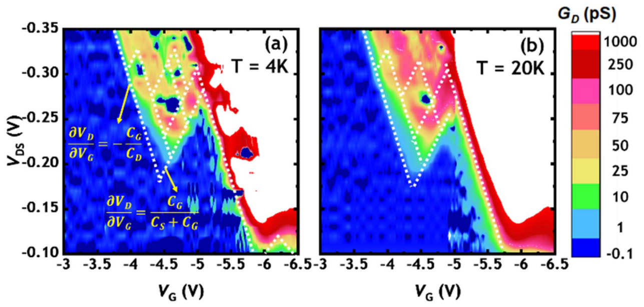

**FIGURE 6. Contour plot of $G_D$-$V_D$-$V_G$ characteristics of Ge-QD SHTs measured at T = (a) 4 K and (b) 20 K.**

from electron-electron/hole-hole interactions and the excitation energy (ΔE) of the QD with a constant number (N) of electrons or holes (that is, the energy-level spacing between $E_{N+1}$ and $E_N$). The addition energy is proportional to the gate-voltage spacings ($\Delta V_G$) between Coulomb oscillatory current peaks in a form of $E_{add} = E_C + \Delta E = \alpha \Delta V_G$ [21].

For a large QD with many electrons/holes, Coulomb oscillations are usually periodic (that is, $\Delta V_G$ between the oscillatory current peaks is a constant) because ΔE caused by weak quantum confinement effects is much smaller than $E_C$ arising from electron-electron or hole-hole interactions, i.e., $\Delta E << E_C$. Therefore, the feature of periodic Coulomb oscillation, which is usually predicted by an orthodox theory, is a consequence of $E_C$ homogeneity and negligible ΔE. In general, such a periodic Coulomb oscillation feature was observed only at very low temperature due to the small $E_C$ for a large QD.

In a small QD containing few charges, both electron-electron (or hole-hole) interactions and quantum confinement effects become sufficiently strong and thereby, both $E_C$ and ΔE are large and comparable in magnitude. Kouwenhoven et al., have reported that ΔE is a function of electron number (N) and highly dependent on the dimensionality. Reference [21] For instance, ΔE for a zero-dimensional QD formed by 3D metals or self-assembled semiconductor nanocrystals is large for small N (that is, few-electron or few-hole regime) and decreases as N increases, following a power law of $\Delta E \propto 1/N^{1/3}$ [21]. Therefore, for SETs/SHTs with a small QD and in few-electron/few-hole regime, the fact of nonuniform spacings between energy levels (unequal ΔE) becoming comparable to the charging energy ($E_C$) breaks the periodicity of the Coulomb blockade oscillations [22], [23], [24], [25], [26], [27].

Our Ge QD is smaller than the Bohr radius of 24.9 nm for Ge and capacitively couples to heavily-doped Si reservoirs via hard-wall barriers of $Si_3N_4$ with a barrier height of > 2 eV and barrier width of 9 nm. The hard-wall barriers of $Si_3N_4$ indeed induce strong quantum confinement effects in our small Ge QD, as evidenced by large (> 49 meV) and unequal addition energies extracted from the aperiodic Coulomb oscillation peaks (Figs. 5 and 6).

Our experimentally-observed NDC phenomenon in Fig. 4 is attributable to combined effects of large energy-level spacings of our small Ge QD [17], [18], [19], [20] and limited densities of states (DOS) full of charges in heavily-doped Si reservoirs. In this work, the doping concentration of $p^+$-Si reservoirs is approximate $5 \times 10^{19} - 1 \times 10^{20}$ cm$^{-3}$ formed by ion implantation of boron with a dose of $1 \times 10^{15}$ cm$^{-2}$ and energy of 17 keV into a 50 nm-thick Si. Calculated values of half-width (δ) of the dopant DOS in Si are 4 – 8 meV for boron concentration of $N_{dop} \sim 5 \times 10^{19} - 1 \times 10^{20}$ cm$^{-3}$, using the equation of $\delta = rN_{dop}{}^{1/2}\,[1 - \exp(-s/N_{dop})]$ given by [28] with $r = 4.2 \times 10^{-12}$ eV cm$^{-3/2}$ and $s = 10^{19}$ cm$^{-3}$. Thus, the narrow bandwidth (8–16 meV) of the dopant DOS in our Si reservoir is insufficient to cover more than two resonant energy levels in our 20 nm Ge QD whose discrete energy levels are well separated in large spacings.

In contrast to small PVCR of SETs/SHTs with gate-defined Si/Ge QDs based on Si/SiGe or SiGe/Ge two-dimensional electron/hole gas (2DEG/2DHG) heterostructures having weak, soft-wall confinement, [8], [29], [30], [31], [32] our self-organized Ge QD/$Si_3N_4$ SHTs indeed exhibit oscillatory current peaks with high PVCR of > 200 and > 30 at T = 4 K and 20 K, respectively, thanks to strong quantum confinement effect in our small Ge QD caused by hard-wall tunnel barriers of $Si_3N_4$. While the large tunneling resistance ($10^{13}$–$10^{14}$ Ω) of our hard-wall $Si_3N_4$ barriers indeed effectively reduces the background quantum leakage to ≤10 fA, the resulting tunneling current is small in magnitude (pA – tens pA) as well. It is a known fact that tunneling rates and tunneling resistances are essentially determined by the height and width of tunneling barriers. We envisage to increase tunneling current from pA – tens pA to sub-nA – nA by reducing the sidewall thickness of $Si_3N_4$ overlayers from 10 nm to 5 nm. Additionally, we envisage that both PVCRs and operating temperature of our Ge-QD SHTs could be further increased by reducing the Ge QD size, which is controllably achieved by reducing the geometrical conditions of the SiGe spacer island by adjusting the process times of deposition and etch back.

Fig. 4 shows a relatively large Coulomb gap of hundred mV or close to 1 V in our Ge-QD SHTs. The large drain voltage required for activating charge tunneling is possibly due to the large work-function difference of 1.2 eV between $p^+$-Si S/D reservoirs ($\phi_{p+-Si} \sim 5.15$ eV) and the Ge QD ($\phi_{Ge} \sim 4.0$ eV). The work-function difference could be reduced by converting the $p^+$-Si reservoirs to $Ni_xSi$ reservoirs ($\phi_{NiSi} \sim 4.3 - 4.6$ eV) using self-aligned silicidation (Salicide) processes, which is a prevailing technology for fabricating metal-like S/D electrodes in CMOS transistors. The metallic S/D electrodes will also provide full bandwidth of charge reservoirs for producing current staircases.

## V. CONCLUSION

We have advanced the state-of-the-art for the fabrication of Ge-QD SHTs with self-organized tunnel barriers and self-aligned electrodes using an ingenious combination of lithographic patterning, sidewall-spacer technique, and self-assembled growth. The self-aligned electrodes do indeed suppress the gate overlap of the S/D electrodes

thereby improving the Coulomb oscillatory current with higher PVCRs. Our Ge QD SHTs feature aperiodic oscillatory current and NDC behaviors within the temperature range of 4 – 40 K with corresponding estimated addition energies > 49 meV for few holes regime.

Thanks to large addition energies and well-separated energy levels, our small Ge QDs with few-charges are desirable for many applications including metrology, electrometry, and quantum registers from technological perspectives. References [22], [25] For instance, Horibe et al. [26] have reported that to implement quantum logic gates based on electron spin, it is necessary to reduce the electron number in individual QDs to levels of a few-electrons or even a single-electron to create spin states that are energetically well defined and separated from other states.

## ACKNOWLEDGMENT

The authors would like to thank the technical support of Taiwan Semiconductor Research Institute (TSRI) on the cryogenic measurements.

## REFERENCES

[1] D. V. Averin and K. K. Likharev, "Coulomb blockade of single-electron tunneling, and coherent oscillations in small tunnel junctions," *J. Low Temp. Phys.*, vol. 62, no. 3, pp. 345–372, Feb. 1986, doi: 10.1007/BF00683469.

[2] S. Lee, Y. Lee, E. B. Song, and T. Hiramoto, "Observation of single electron transport via multiple quantum states of a silicon quantum dot at room temperature," *Nano Lett.*, vol. 14, no. 1, pp. 71–77, Jan. 2014, doi: 10.1021/nl403204k.

[3] S. J. Shin et al., "Room-temperature charge stability modulated by quantum effects in a nanoscale silicon island," *Nano Lett.*, vol. 11, no. 4, pp. 1591–1597, Apr. 2011, doi: 10.1021/nl1044692.

[4] A. Rossi et al., "An accurate single-electron pump based on a highly tunable silicon quantum dot," *Nano Lett.*, vol. 14, no. 6, pp. 3405–3411, Jun. 2014, doi: 10.1021/nl500927q.

[5] E. Prati et al., "Few electron limit of n-type metal oxide semiconductor single electron transistors," *Nanotechnology*, vol. 23, no. 21, May 2012, Art. no. 215204, doi: 10.1088/0957-4484/23/21/215204.

[6] B. Voisin et al., "Few-electron edge-state quantum dots in a silicon nanowire field-effect transistor," *Nano Lett.*, vol. 14, no. 4, pp. 2094–2098, Apr. 2014, doi: 10.1021/nl500299h.

[7] Y. Maeda, N. Tsukamoto, Y. Yazawa, Y. Kanemitsu, Y. Masumoto, "Visible photoluminescence of Ge microcrystals embedded in $SiO_2$ glassy matrices," *Appl. Phys. Lett.*, vol. 59, no. 24, pp. 3168–3170, Aug. 1991, doi: 10.1063/1.105773.

[8] X. Xue et al., "Benchmarking gate fidelities in a Si/SiGe two-qubit device," *Phys. Rev. X*, vol. 9, no. 2, Apr. 2019, Art. no. 21011, doi: 10.1103/PhysRevX.9.021011.

[9] K. Chen, C. Wang, T. George, and P. W. Li, "The pivotal role of SiO formation in the migration and Ostwald ripening of Ge quantum dots," *Appl. Phys. Lett.*, vol. 105, no. 12, Sep. 2014, Art. no. 122102, doi: 10.1063/1.4896504.

[10] T. Huang, K. Peng, C. Chen, H.-C. Lin, T. George, and P.-W. Li, "Tunable diameter and spacing of double Ge quantum dots using highly-controllable spacers and selective oxidation of SiGe," *Sci. Rep.*, vol. 9, Aug. 2019, Art. no. 11303, doi: 10.1038/s41598-019-47806-0.

[11] P. H. Liao, K. P. Peng, H. C. Lin, T. George, and P. W. Li, "Single-fabrication-step Ge nanosphere/$SiO_2$/SiGe heterostructures: A key enabler for realizing Ge MOS devices," *Nanotechnology*, vol. 29, no. 20, Mar. 2018, Art. no. 205601, doi: 10.1088/1361-6528/aab17b.

[12] T. George, P. W. Li, K. Chen, K. Peng, and W. Lai, "'Symbiotic' semiconductors: Unusual and counter-intuitive Ge/Si/O interactions," *J. Phys. D Appl. Phys.*, vol. 50, no. 10, Feb. 2017, Art. no. 105101, doi: 10.1088/1361-6463/aa59ab.

[13] I.-H. Wang, P.-Y. Hong, K.-P. Peng, H.-C. Lin, T. George, and P.-W. Li, "Germanium quantum-dot array with self-aligned electrodes for quantum electronic devices," *Nanomaterials*, vol. 11, no. 10, pp. 2743–2757, Nov. 2021, doi: 10.3390/nano11102743.

[14] R. C. Pan, I. H. Wang, C. C. Lai, T. George, H. C. Lin, and P. W. Li, "Germanium quantum-dot single-hole transistors with self-organized tunnel barriers and self-aligned electrodes using ingenious sidewall spacer and oxidation Techniques," in *Proc. 6th IEEE Electron Devices Technol. Manuf. Conf. (EDTM)*, 2022, pp. 321–323, doi: 10.1109/EDTM53872.2022.9798091.

[15] I.-H. Chen, K.-H. Chen, W.-T. Lai, and P.-W. Li, "Single germanium quantum-dot placement along with self-aligned electrodes for effective management of single charge tunneling," *IEEE Trans. Electron Devices*, vol. 59, no. 12, pp. 3224–3230, Dec. 2012, doi: 10.1109/TED.2012.2217973.

[16] I. H. Chen, W. T. Lai, and P. W. Li, "Realization of solid-state nanothermometer using Ge quantum-dot single-hole transistor in few-hole regime," *Appl. Phys. Lett.*, vol. 104, no. 24, Jun. 2014, Art. no. 243506, doi: 10.1063/1.4884296.

[17] Y. Ono and Y. Takahashi, "Observation and circuit application of negative differential conductance in silicon single-electron transistors," *Jpn. J. Appl. Phys.*, vol. 41, no. 4S, pp. 2569–2573, Apr. 2002, doi: 10.1143/JJAP.41.2569.

[18] H. Ishikuroa and T. Hiramoto, "Quantum mechanical effects in the silicon quantum dot in a single-electron transistor," *Appl. Phys. Lett.*, vol. 71, no. 25, pp. 3691–3693, Dec. 1997, doi: 10.1063/1.120483.

[19] K. Miyaji, M. Saitoh, and T. Hiramoto, "Compact analytical model for room-temperature-operating silicon single-electron transistors with discrete quantum energy levels," *IEEE Trans. Nanotechnol.*, vol. 5, no. 3, pp. 167–173, May 2006, doi: 10.1109/TNANO.2006.869949.

[20] B. Pruvost, H. Mizuta, and S. Oda, "Voltage-limitation-free analytical single-electron transistor model incorporating the effects of spin-degenerate discrete energy states," *J. Appl. Phys.*, vol. 103, no. 5, Mar. 2008, Art. no. 54508, doi: 10.1063/1.2838491.

[21] L. P. Kouwenhoven, C. M. Marcus, P. L. McEuen, S. Tarucha, R. M. Westervelt, and N. S. Wingreen, "Electron transport in quantum dots," in *Mesoscopic Electron Transport*. Dordrecht, The Netherlands: Springer, 1997, pp. 105–214, doi: 10.1007/978-94-015-8839-3_4.

[22] L.P. Kouwenhoven *et al.*, "Excitation spectra of circular, few-electron quantum dots," *Science*, vol. 278, no. 5344, pp. 1788–1792, Dec. 1997, doi: 10.1126/science.278.5344.1788.

[23] L. P. Kouwenhoven, D. G. Austing, and S. Tarucha, "Few-electron quantum dots," *Rep. Prog. Phys.*, vol. 64, no. 6, pp. 701–736, 2001, doi: 10.1088/0034-4885/64/6/201.

[24] S. Tarucha, D. G. Austing, T. Honda, R. J. van der Hage, and L. P. Kouwenhoven, "Shell filling and spin effects in a few electron quantum dot," *Phys. Rev. Lett.*, vol. 77, no. 17, pp. 3613–3616, Oct. 1996, doi: 10.1103/PhysRevLett.77.3613.

[25] S. K. Shin, S. Huang, N. Fukata, and K. Ishibashi, "Top-gated germanium nanowire quantum dots in a few-electron regime," *Appl. Phys. Lett.*, vol. 100, no. 7, Feb. 2012, Art. no. 73103, doi: 10.1063/1.3684941.

[26] K. Horibe, T. Kodera, and S. Oda, "Lithographically defined few-electron silicon quantum dots based on a silicon-on-insulator substrate," *Appl. Phys. Lett.*, vol. 106, no. 8, Feb. 2015, Art. no. 83111, doi: 10.1063/1.4913321.

[27] W. H. Lim et al., "Observation of the single-electron regime in a highly tunable silicon quantum dot," *Appl. Phys. Lett.*, vol. 95, no. 24, Dec. 2009, Art. no. 242102, doi: 10.1063/1.3272858.

[28] P. P. Altermatt, A. Schenk, B. Schmithüsen, and G. Heiser, "A simulation model for the density of states and for incomplete ionization in crystalline silicon. II. Investigation of Si:As and Si:B and usage in device simulation," *J. Appl. Phys.*, vol. 100, no. 11, Dec. 2006, Art. no. 113715, doi: 10.1063/1.2386935.

[29] W. Lawrie et al., "Quantum dot arrays in silicon and germanium," *Appl. Phys. Lett.* vol. 116, no. 8, Feb. 2020, Art. no. 80501.

[30] S. Angus, A. Ferguson, A. Dzurak, and R. Clark, "Gate-defined quantum dots in intrinsic silicon," *Nano Lett.*, vol. 7, no. 7, pp. 2051–2055, Jul. 2007, doi: 10.1021/nl070949k.

[31] T. Takakura et al., "Single to quadruple quantum dots with tunable tunnel couplings," *Appl. Phys. Lett* vol. 104, no. 11, Mar. 2014, Art. no. 113109, doi: 10.1063/1.4869108.

[32] F. N. M. Froning et al., "Single, double, and triple quantum dots in Ge/Si nanowires," *Appl. Phys. Lett.*, vol. 113, no. 7, May 2018, Art. no. 73102, doi: 10.1063/1.5042501.
